# Association of the dietary index for gut microbiota and dietary inflammation index with metabolic dysfunction-associated steatotic liver disease and metabolic alcohol-associated liver disease

**DOI:** 10.3389/fimmu.2025.1593245

**Published:** 2025-07-17

**Authors:** Wenhao Wu, Zebin Hou

**Affiliations:** ^1^ The Second Clinical Medical School, SHanxi Medical University, Shanxi, China; ^2^ Department of Thyroid Surgery, Shanxi Provincial People’s Hospital Affiliated to Shanxi Medical University, Taiyuan, Shanxi, China

**Keywords:** dietary index for gut microbiota, dietary inflammatory index, metabolic dysfunction-associated steatotic liver disease, alcohol-associated metabolic dysfunction-associated liver disease, inflammation, metabolic dysfunction

## Abstract

**Background:**

Metabolic dysfunction-associated steatotic liver disease (MASLD) and alcohol-associated metabolic dysfunction-associated liver disease (MetALD) are significant public health concerns, with diet playing a pivotal role in their pathogenesis. Aims: Using data from the National Health and Nutrition Examination Survey (NHANES) 2007–2018. This study investigates the associations of the dietary index for gut microbiota (DI-GM), dietary inflammatory index (DII), and their combined effects with MASLD/MetALD, while exploring the mediating roles of inflammation and metabolic dysfunction.

**Methods:**

Data from the 2007 to 2018 NHANES included 9,529 participants. DI-GM and DII were calculated using 24-hour dietary recalls. Inflammatory and metabolic biomarkers—including triglyceride-glucose (TyG) index, metabolic score (MS), C-reactive protein (CRP), systemic immune inflammation index (SII), and systemic inflammatory response index (SIRI)—were analyzed. Multivariable logistic and linear regression, subgroup analyses, and restricted cubic spline (RCS) models assessed associations and dose-response relationships. Mediation analysis evaluated the roles of inflammatory and metabolic markers.

**Results:**

Higher DI-GM scores were significantly associated with reduced MASLD (OR = 0.59, 95% CI: 0.46–0.75) and MetALD (OR = 0.57, 95% CI: 0.46–0.70). Conversely, higher DII scores were positively associated with MASLD (OR = 1.57, 95% CI: 1.23–2.01) and MetALD (OR = 1.40, 95% CI: 1.13–1.75). DI-GM was inversely associated with inflammation and metabolic markers (TyG: β= -0.05, MS: β= -0.11, CRP: β= -0.12, SII: β= -0.08, SIRI: β= -0.09), while DII exacerbated these markers (TyG: β= 0.06, MS: β= 0.18, CRP: β=0.14, SII: β= 0.11, SIRI: β= 0.10). The combined effects of DI-GM and DII further demonstrated that a gut microbiota-healthy and anti-inflammatory diet synergistically reduced MASLD (OR = 0.59, 95% CI: 0.43–0.81) and MetALD risks (OR = 0.58, 95% CI: 0.44–0.76). Mediation analysis confirmed that inflammation and metabolism significantly mediated the diet-disease associations (p < 0.05).

**Conclusion:**

Higher DI-GM and lower DII are associated with reduced MASLD/MetALD risks, partially mediated by alleviating systemic inflammation and metabolic dysfunction. These findings highlight dietary interventions targeting gut microbiota and inflammation as strategies for early prevention of MASLD and MetALD.

## 1 Introduction

Metabolic dysfunction-associated steatotic liver disease (MASLD), previously known as non-alcoholic fatty liver disease (NAFLD), has evolved from a rare medical condition to the most prevalent chronic liver disease globally, imposing a significant medical and economic burden (1–3). Historically, NAFLD was diagnosed based on exclusion criteria, with individuals consuming alcohol above minimal thresholds (≥20 g/d for women and ≥30 g/d for men) often excluded, even if they exhibited metabolic risk factors (4). However, the Delphi consensus in 2023 introduced a revised nomenclature, establishing a universal term—steatotic liver disease (SLD)—to encompass all patients with evidence of hepatic steatosis, with refined subclassifications such as MASLD and alcohol-associated metabolic dysfunction-associated liver disease (MetALD) to more accurately reflect the interplay of metabolic and alcohol-related risk factors (5). Given the intricate ties between MASLD, metabolic dysregulation, and nutritional intake, medical nutrition therapy (MNT) has emerged as a cornerstone strategy for managing this condition (6).

Gut microbiota play a critical role in the development of MASLD and MetALD, with research indicating that several dietary components influencing gut microbiota correlate with SLD progression (7). To assess the contribution of daily dietary intake to gut microbiota health maintenance, Bezawit E. Kase et al. conducted a systematic review of interventional and prospective studies, identifying 14 foods and nutrients with beneficial or detrimental effects on gut microbiota (8). These components were used to develop the scientifically validated dietary index for gut microbiota (DI-GM), which evaluates the potential of dietary intake to influence gut microbiota diversity and functionality (8). The beneficial foods included in DI-GM are associated with increased gut microbial α-diversity and β-diversity indices, elevated short-chain fatty acids (SCFAs) such as butyrate, acetate, propionate, and isobutyrate, and shifts in microbial community composition (e.g., Bacteroidetes/Firmicutes ratio) (9–12). Conversely, the detrimental foods are linked to reduced microbiota diversity and unfavorable microbial profiles. DI-GM correlated positively with urinary enterolignans, biomarkers of gut microbiota diversity, further validating its utility as a standardized tool for assessing diet-microbiome interactions. This index facilitates interdisciplinary collaboration across nutrition, microbiology, medicine, and epidemiology, enabling the evaluation of gut microbiota diversity and health outcomes through dietary patterns. In a cross-sectional study involving 7,243 participants, a significant negative linear association was observed between DI-GM and MASLD prevalence, indicating the potential protective role of diet-induced gut microbiota modulation against liver steatosis (13). However, the specific relationships between DI-GM and the newly designated MASLD/MetALD remain to be elucidated, along with the underlying mechanisms linking dietary patterns to liver disease progression.

Inflammation is a critical mechanism underlying the pathogenesis of MASLD and MetALD (6). Research has demonstrated that various nutrients with anti-inflammatory properties, such as vitamin C and dietary fiber, and pro-inflammatory components, such as carbohydrates and fats, are closely associated with liver health (6). Dietary inflammatory index (DII), a scientifically validated metric, evaluates dietary inflammatory potential by aggregating the intake of nutrients with divergent anti-inflammatory or pro-inflammatory characteristics (14). While some studies have explored the relationship between DII and MASLD, the results remain inconsistent, and research on the association between DII and MetALD is currently lacking (15–18). Therefore, a more comprehensive investigation is warranted to elucidate the specific role of anti-inflammatory diets in the progression and development of MASLD and MetALD.

Insulin resistance, disordered glucose and lipid metabolism, and inflammation have been demonstrated to contribute to hepatic steatosis and lipid accumulation in the liver (19).Triglyceride-glucose (TyG), as a clinically useful surrogate marker, has been shown to outperform individual glucose and triglyceride levels in assessing metabolic dysfunction. Furthermore, TyG demonstrates greater accuracy in diagnosing metabolic syndrome compared to the homeostatic model assessment of insulin resistance (HOMA-IR) (20). The development of a metabolic score (MS) provides a valuable tool for identifying individuals with systemic metabolic abnormalities (20). Given the advantages of the TyG and MS indices in terms of accessibility and applicability in the general population, they are frequently employed as indicators of metabolic dysfunction (21, 22). Additionally, systemic inflammatory markers such as C-reactive protein (CRP), serum inflammatory response index (SIRI), and systemic immune inflammation index (SII) are critical in evaluating the body’s response to inflammation (23, 24). Importantly, the detrimental effects of these indices—TyG, CRP, SIRI, and SII—on the progression of MASLD have been well-documented in previous studies (25, 26). Therefore, this study selected these indices as potential mediators of the association between metabolic dysfunction and MASLD pathogenesis.

Diet quality plays a pivotal role in modulating and preventing metabolic disturbances and inflammation. Previous studies have demonstrated that a higher DII is associated with an increased risk of metabolic syndrome (27, 28), as chronic inflammation often accompanies metabolic dysfunction (29). While the relationship between DI-GM and metabolic disorders remains understudied, research has shown that maintaining a healthy gut microbiome equilibrium can effectively reverse dysregulation in glucose and lipid metabolism (30, 31). Importantly, inflammation and gut microbiome dysregulation are mutually reinforcing processes, with inflammation disrupting gut microbiome stability and dysregulated microbiota producing endotoxins that exacerbate inflammation (19). These findings underscore the necessity of investigating the combined effects of DI-GM and DII on metabolic disorders and inflammation. Furthermore, considering the association between metabolic dysfunction, inflammation, and SLD, we hypothesize that there is an interconnected relationship among DI-GM, DII, metabolic dysfunction indices (TyG, MS), inflammatory markers (CRP, SIRI, SII), and the development of MASLD and MetALD. Specifically, the potential mediating effects of metabolic dysfunction and systemic inflammation in the dietary quality-MASLD/MetALD association warrant further exploration.

This study leverages the representative sample data from the National Health and Nutrition Examination Survey (NHANES) to elucidate the associations between diet quality (joint exposure of DI-GM and DII) and key indicators of metabolic dysfunction (TyG, MS), systemic inflammatory markers (CRP, SIRI, SII), MASLD, and MetALD. Furthermore, it aims to clarify the mediating roles of metabolic dysfunction and inflammation in the relationship between diet quality and MASLD/MetALD progression.

## 2 Method

### 2.1 Data sources

**Figure 1:**
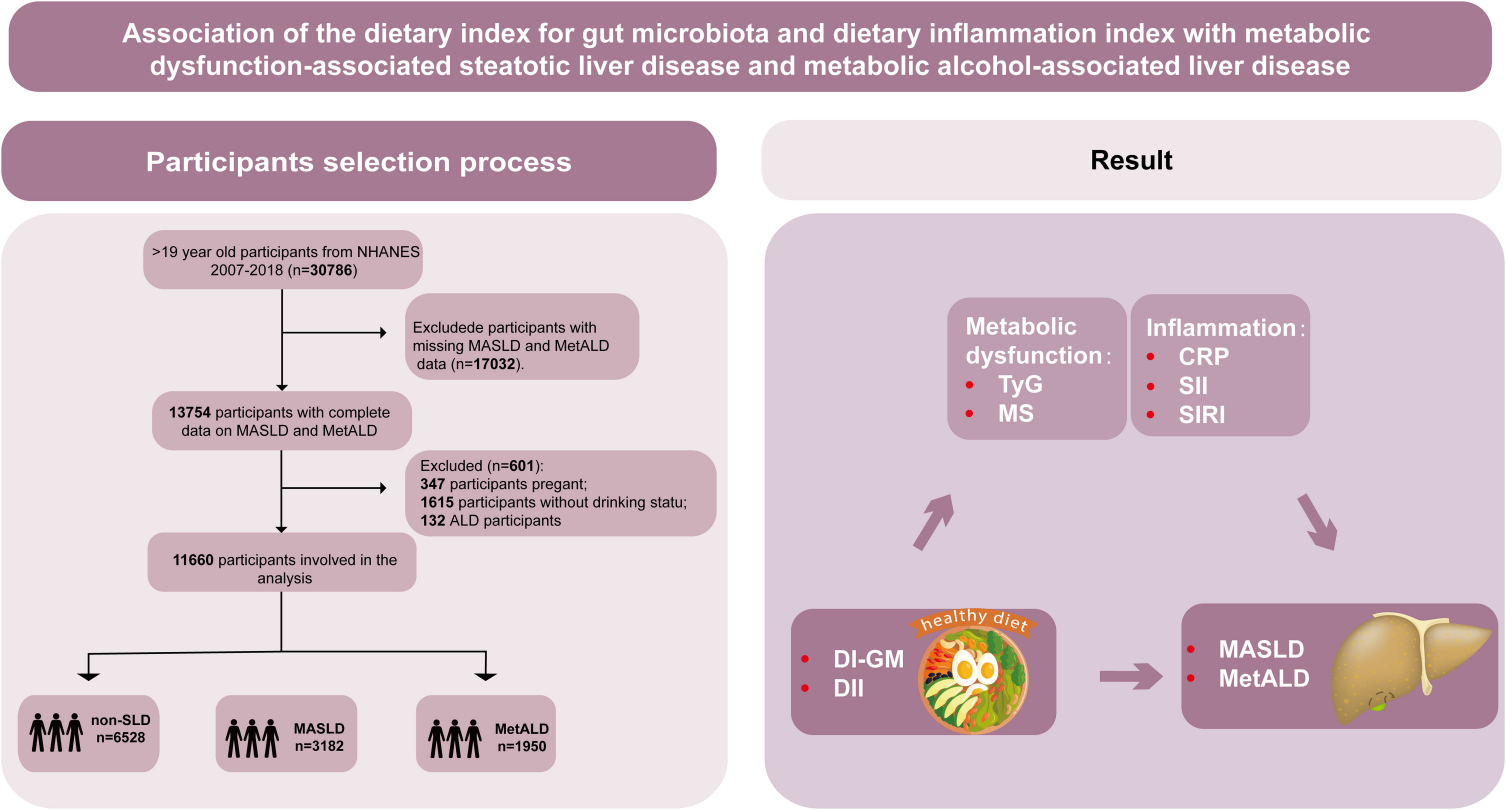
Graphical abstract and flowchart of studied participants selection. NHANES, National Health and Nutrition Examination Survey; DI-GM, dietary index for gut microbiota; DII, dietary inflammation index; MASLD, metabolic dysfunction-associated steatotic liver disease; MetALD, metabolic dysfunction and alcohol-associated liver disease; Non-SLD, no steatotic liver disease; TyG, triglyceride-glucose index; MS, metabolic score; CRP, C-reactive protein; SII, systemic immune-inflammation index; SIRI, systemic inflammatory response index.

NHANES is a national survey in the United States that evaluates the health and nutritional status of non-institutionalized civilians, integrating interview and physical examination data. Methodological details are described in a separate publication (32). The survey, which was approved by the National Center for Health Statistics (NCHS) ethics committee and conducted in accordance with the STROBE guidelines, encompasses demographic characteristics, socioeconomic status, dietary habits, and health conditions (33). The study population initially included 30,786 individuals aged ≥20 years in 2007–2018. Exclusions were made for the following reasons: pregnancy (n=347), missing MASLD/MetALD data (n=17,032), and missing alcohol consumption data (n=1,615). After exclusions, 11,792 participants remained. Among these, 6,528 (56%) individuals did not have suspected liver disease (SLD), 3,182 (27.3%) had MASLD, 1,950 (16.7%) had MetALD, and 132 (1.1%) had alcoholic liver disease (ALD). Due to the small number of ALD cases, these participants were excluded, and the study focused on MASLD and MetALD, resulting in a final sample size of 11,660 (**Figure 1**).

### 2.2 Dietary index for gut microbiota

Bezawit E. Kase and colleagues identified 14 food or nutrient components associated with gut microbiome health in a systematic review of 106 studies (8). Specifically, these components were classified into beneficial and detrimental categories. The beneficial components included avocado, broccoli, chickpeas, coffee, cranberries, fermented dairy products, fiber, green tea, soy products, and whole grains. In contrast, red meat, processed meats, refined grains, and high-fat diets (where fat contributes ≥40% of energy intake) were categorized as detrimental components. For beneficial foods, participants whose intake exceeded the sex-specific median were assigned a score of 1, while those below the median were assigned 0. For detrimental components, participants with intake above the sex-specific median were assigned 0, while those below the median were assigned 1 (with the exception of high-fat diets, which were scored using a fixed threshold of 40% energy from fat). The DI-GM score was calculated as the sum of the scores for each component, resulting in a score range of 0 to 14. In this study, participants in the highest tertile of DI-GM scores were categorized as consuming a “gut microbiota-healthy diet,” while those in the lowest tertile were categorized as consuming a “gut microbiota-unhealthy diet.”

### 2.3 Dietary inflammatory index

DII is a tool designed to assess the potential inflammatory risk of an overall diet. It is based on extensive literature, including animal and epidemiological studies, to evaluate the inflammatory potential of dietary patterns (14). A diet with pro-inflammatory effects refers to its capacity to significantly elevate circulating levels of pro-inflammatory cytokines, such as interleukin-1β (IL-1β), interleukin-6 (IL-6), tumor necrosis factor-alpha (TNF-α), or CRP, or decrease levels of anti-inflammatory cytokines like interleukin-4 (IL-4) and interleukin-10 (IL-10). The DII score for each individual is calculated based on data from a 24-hour dietary recall interview, which allows for the assessment of the diet’s inflammatory potential. Food parameters included both anti-inflammatory components (alcohol, vitamin B-6, β-carotene, caffeine, cinnamaldehyde, dietary fiber, folate, garlic, ginger, magnesium, monounsaturated fatty acids, niacin, n-3 fatty acids, n-6 fatty acids, onions, polyunsaturated fatty acids, thiazole, tafron, selenium, thiamine, curcumin, vitamin A, vitamin D, vitamin C, vitamin E, zinc, green tea/black tea, flavan-3-ol, flavones, flavonols, flavonoids, anthocyanins, isoflavones, chili, thyme/oregano, and rosemary) and pro-inflammatory components (cholesterol, carbohydrates, energy, fat, iron, vitamin B12, protein, and saturated fat). In this study, participants in the highest tertile of DII scores were categorized as consuming a pro-inflammatory diet, while those in the lowest tertile were considered to follow an anti-inflammatory diet.

### 2.4 Combined dietary pattern classification

To evaluate synergistic effects of inflammatory potential and gut microbiota modulation, a combined dietary variable was developed by integrating DI-GM and DII tertiles. Participants were stratified into three mutually exclusive groups based on their dietary patterns. Those in the first DI-GM tertile (T1) combined with the third DII tertile (T3) were classified as following a pro-inflammatory and gut microbiota-unhealthy diet. Individuals in the DI-GM T3 and DII T1 were categorized as adhering to an anti-inflammatory and gut microbiota-healthy diet. All remaining participants not meeting these thresholds comprised the intermediate dietary pattern group.

### 2.5 MASLD and MetALD definitions

Hepatic steatosis was diagnosed using the Fatty Liver Index (FLI), with FLI ≥ 60 indicating a high probability of SLD and FLI < 60 representing low risk (34). Participants were excluded if they had viral hepatitis, autoimmune liver disease, genetic liver disorders, drug-induced liver injury, or alcohol-related liver disease, or if they reported excessive alcohol intake (≥30 g/day for males or ≥20 g/day for females) based on 24-hour dietary recalls using the US department of agriculture (USDA) automated multiple-pass method (35). MASLD was defined as SLD concurrent with at least one of the following cardiometabolic risk criteria:

1. Body mass index (BMI) ≥25 kg/m² or waist circumference (WC) ≥94 cm (males)/≥80 cm (females).
2. Fasting blood glucose (FBG) ≥100 mg/dL, 2-hour post-load glucose ≥140 mg/dL, hemoglobin A1c ≥5.7%, diabetes mellitus diagnosis, or hypoglycemic therapy use.
3. Blood pressure ≥130/85 mmHg or antihypertensive medication use.
4. Fasting plasma triglycerides ≥150 mg/dL or lipid-lowering treatment.
5. High-density lipoprotein cholesterol (HDL-C) <40 mg/dL (males) or <50 mg/dL (females) or lipid-lowering therapy (5).

Participants with SLD and moderate alcohol consumption (20–50 g/day for females or 30–60 g/day for males) alongside ≥1 cardiometabolic criterion were classified as MetALD. Those exceeding these alcohol thresholds (>50 g/day for females or >60 g/day for males) were categorized as ALD, irrespective of cardiometabolic status (4).

### 2.6 Metabolic dysfunction indicators

Blood pressure measurements were conducted at the mobile examination center (MEC). Fasting glucose, triglycerides, total cholesterol, and uric acid levels were analyzed using automated biochemical analyzers, with detailed protocols available on the NHANES website (https://www.cdc.gov/nchs/nhanes/index.htm). Mean arterial pressure (MAP) was calculated as diastolic pressure plus one-third of the difference between systolic and diastolic pressure (20). TyG index was derived as the natural logarithm of fasting triglyceride levels (mg/dL) multiplied by fasting glucose levels (mg/dL), divided by two (20). A metabolic score (MS) was constructed by summing z-transformed values of total cholesterol, uric acid, MAP, and TyG index (20).

### 2.7 Systemic inflammatory indices

CRP levels were quantified using high-sensitivity assays. SIRI was calculated as the product of monocyte count and neutrophil count divided by lymphocyte count (36). SII was calculated as the product of platelet count and neutrophil count divided by lymphocyte count (36). All hematological parameters were measured using standardized protocols to ensure analytical consistency.

### 2.8 Covariate assessment

Potential confounding factors were also collected as covariates, including age, sex, race/ethnicity (mexican American, non-Hispanic white, non-Hispanic black, other race), education level (high school or higher, less than high school), marital status (married, unmarried), and poverty income ratio (PIR), prediabetes mellitus (preDM), history of cardiovascular diseases (CVD), energy intake, physical activity and smoking status. The poverty income ratio (PIR), which reflects the family’s socioeconomic status, was calculated by dividing the family income by the poverty guidelines corresponding to the participant’s household size, adjusted for the year and state of residence (37). PIR levels were stratified into three categories: low (PIR < 1.3), middle (1.3 ≤ PIR ≤ 3.5), and high (PIR > 3.5). Prediabetes mellitus (preDM) was defined as FBG levels between 100–125 mg/dL (5.6–6.9 mmol/L) or hemoglobin A1c (HbA1c) levels between 5.7%–6.4% (39–47 mmol/mol). Diabetes was diagnosed based on FBG ≥ 126 mg/dL, HbA1c ≥ 6.5%, or self-reported physician diagnosis and/or antidiabetic treatment (38). CVD included myocardial infarction, congestive heart failure, angina, coronary artery disease, and stroke (38). Physical activity was assessed based on a detailed physical activity questionnaire as described previously (39). Total energy intake was computed using three-day dietary recall food composition tables (39). Smoking status was defined as “smoker” (lifetime smoking ≥ 100 cigarettes) or “non-smoker” (20).Alcohol consumption was measured in grams per day (g/d), calculated as the average daily alcohol intake based on the question: “On average, how many alcoholic drinks did you/he/she have in the past 12 months?” According to U.S. standards, one standard alcoholic drink contains 14 g of alcohol (40). Other physical and blood measurements were obtained by trained medical personnel using standardized protocols in the laboratory.

### 2.9 Statistical analysis

According to the guidelines of NCHS, appropriate weighting was applied to all analytical procedures. Continuous variables were expressed as weighted means (± standard deviation [SD]), while categorical variables were presented as frequencies (proportions), with weighted ANOVA and chi-square tests employed for comparisons. Weighted multivariate linear regression analyses across three hierarchical models were utilized to assess associations between the DI-GM, DII, their combinations, and intermediary factors. Similarly, multivariable logistic regression models evaluated relationships between DII, DI-GM, and their combinations with MASLD/MetALD risk in 3 models. Linear trends were examined by assigning median values to DI-GM/DII tertiles and modeling these as continuous variables. The crude model remained unadjusted, Model 1 adjusted for age and sex, while Model 2 further adjusted for race/ethnicity, education level, marital status, PIR, prediabetes/diabetes, CVD, smoking status, energy intake and physical activity. Following comprehensive covariate adjustment, restricted cubic spline (RCS) analysis was conducted to examine linear and nonlinear relationships between DI-GM/DII and MASLD/MetALD risk, with node selection optimized through minimization of Akaike’s Information Criterion (AIC) (41). Mediation analysis using the PROCESS tool with 1000 bootstrap resamples evaluated potential mediating roles of TyG, MS, CRP, SII, and SIRI in the DII/DI-GM-MASLD/MetALD associations, with mediation established when statistically significant relationships existed between exposures and mediators (X-M) and between mediators and outcomes (M-Y) (42). Stratified analyses examined effect modification by age (<65 vs ≥65 years), race, smoking status, and comorbidities (diabetes/CVD). All RCS, mediation, and stratified analyses were conducted using fully adjusted model 3. Statistical computations were performed in R (version 4.1.2), with two-tailed p-values <0.05 considered statistically significant.

## 3 Result

### 3.1 Characteristics of participants at baseline

The baseline characteristics of the study participants, categorized by DI-GM, DII, and their combinations, are presented in **Table 1**. Overall, participants with a gut microbiota-unhealthy and/or pro-inflammatory diet were older and exhibited a higher prevalence of MASLD, MetALD, diabetes, and CVD compared to those who consumed a gut microbiota-healthy and/or anti-inflammatory diet. Additionally, these individuals demonstrated elevated insulin resistance, as indicated by the TyG, and higher levels of inflammatory markers, including MS, CRP, SII and SIRI. They also had lower educational attainment, income and daily energy intake, and were more likely to be male, married, and current smokers.

**Table 1:** Participants’ characteristics according to DI-GM tertiles, DII tertiles, and different combinations of DIGM and DII.

Characteristic	Total	DI-GM				DII				Different combinations of DI-GM and DII			
		<=4 (N=4477)	4-6 (N=4821)	>=6 (N=2362)	p-value	<=0.81 (N=3886)	0.81-2.66 (N=3887)	>=2.66 (N=3886)	p-value	Gut microbiota-unhealthy and pro-inflammatory(N=2108)	Composite diet category(N=8239)	Gut microbiota-healthy and anti-inflammatory (N=1313)	p-value
MASLD^b^					< 0.0001				0.003				< 0.0001
MASLD	3182(26.51)	1265(27.70)	1304(26.87)	613(23.94)		990(24.97)	1070(26.97)	1122(27.83)		622(28.59)	2245(26.87)	315(21.98)	
MetALD	1950(16.50)	862(19.70)	807(16.51)	281(11.41)		602(15.17)	646(16.14)	702(18.48)		415(19.95)	1383(16.75)	152(10.88)	
Non-SLD	6528(56.99)	2350(52.60)	2710(56.62)	1468(64.65)		2295(59.86)	2170(56.89)	2063(53.69)		1071(51.47)	4611(56.39)	846(67.14)	
**TyG** ^a^	8.58(0.01)	8.61 (0.01)	8.59 (0.01)	8.52 (0.02)	< 0.001	8.56 (0.02)	8.58 (0.02)	8.61 (0.01)	0.048	8.62 (0.02)	8.59 (0.01)	8.49 (0.02)	< 0.001
**MS** ^a^	-0.14(0.04)	-0.09 (0.05)	-0.05 (0.06)	-0.36 (0.06)	< 0.001	-0.20 (0.05)	-0.07 (0.06)	-0.14 (0.06)	0.25	-0.14 (0.08)	-0.07 (0.05)	-0.49 (0.08)	< 0.0001
**CRP** ^a^	0.37(0.01)	0.43 (0.02)	0.36 (0.02)	0.31 (0.02)	0.004	0.29 (0.02)	0.36 (0.02)	0.48 (0.03)	< 0.0001	0.50 (0.03)	0.36 (0.01)	0.28 (0.03)	< 0.001
**SII** ^a^	6.11(0.01)	6.13 (0.01)	6.11 (0.01)	6.08 (0.01)	0.03	6.06 (0.01)	6.12 (0.01)	6.15 (0.01)	< 0.0001	6.16 (0.02)	6.11 (0.01)	6.05 (0.02)	< 0.0001
**SIRI** ^a^	1.18(0.01)	1.22 (0.02)	1.16 (0.01)	1.14 (0.02)	0.01	1.15 (0.02)	1.18 (0.02)	1.21 (0.02)	0.03	1.24 (0.02)	1.17 (0.01)	1.14 (0.03)	0.004
**Age** ^a^	48.50(0.27)	46.81 (0.34)	48.43 (0.36)	51.33 (0.52)	< 0.0001	48.68 (0.39)	48.44 (0.41)	48.36 (0.39)	0.8	47.83 (0.53)	48.16 (0.31)	51.25 (0.62)	< 0.0001
**Male** ^b^	5539(47.38)	2275 (51.55)	2248 (46.36)	1016 (42.70)	< 0.0001	2300 (59.40)	1806 (45.37)	1433 (35.35)	< 0.0001	852 (38.71)	4002 (48.51)	685 (51.75)	< 0.0001
Race and ethnicity^b^					< 0.0001				< 0.0001				< 0.0001
Mexican American	1734 (8.04)	715 (9.50)	762 (8.36)	257 (5.12)		677 (9.10)	549 (7.65)	508 (7.22)		300 (8.32)	1275 (8.40)	159 (5.72)	
Non-Hispanic Black	2323 (10.09)	1108 (13.57)	888 (9.15)	327 (6.34)		590 (6.89)	775 (10.39)	958 (13.55)		597 (16.15)	1564 (9.61)	162 (5.27)	
Non-Hispanic White	5076 (69.19)	1779 (64.31)	2130 (69.97)	1167 (75.44)		1734 (71.62)	1677 (68.43)	1665 (67.13)		842 (64.07)	3586 (69.01)	648 (76.48)	
Other Race	2527 (12.68)	875 (12.61)	1041 (12.53)	611 (13.10)		886 (12.40)	885 (13.53)	756 (12.09)		369 (11.46)	1814 (12.98)	344 (12.53)	
Less than high school^b^	2160 (12.88)	975 (16.43)	870 (11.78)	315 (9.33)	< 0.0001	595 (9.78)	690 (12.50)	875 (16.98)	< 0.0001	510 (19.31)	1495 (12.36)	155 (7.85)	< 0.0001
Married^b^	7016 (62.63)	2482 (57.57)	2959 (63.90)	1575 (68.36)	< 0.0001	2478 (66.47)	2350 (62.29)	2188 (58.51)	< 0.0001	1134 (55.97)	5001 (62.96)	881 (69.18)	< 0.0001
Poverty income ratio level^b^					< 0.0001				< 0.0001				< 0.0001
high	3268 (40.11)	959 (34.56)	1377 (44.11)	932 (54.09)		1425 (52.87)	1077 (42.71)	766 (31.35)		361 (28.22)	2315 (43.05)	592 (60.37)	
low	3282 (19.22)	1458 (25.61)	1375 (20.47)	449 (12.82)		909 (15.83)	1042 (19.56)	1331 (27.40)		761 (29.92)	2297 (20.33)	224 (10.63)	
middle	4113 (34.05)	1671 (39.83)	1660 (35.42)	782 (33.09)		1243 (31.30)	1408 (37.72)	1462 (41.26)		805 (41.86)	2911 (36.62)	397 (29.00)	
CVD^b^	1337 (9.28)	580 (9.88)	525 (9.29)	232 (8.30)	0.21	349 (7.73)	444 (9.33)	544 (11.05)	0.001	314 (11.46)	901 (9.04)	122 (7.95)	0.01
History of Cancer^b^	1122 (10.10)	378 (8.71)	456 (10.15)	288 (12.30)	0.001	389 (10.37)	375 (10.84)	358 (9.02)	0.14	194 (9.29)	773 (10.05)	155 (11.53)	0.26
smoke^b^	2203 (17.87)	1031 (21.58)	882 (18.32)	290 (11.14)	< 0.0001	555 (12.99)	711 (17.03)	937 (24.60)	< 0.0001	541 (25.66)	1521 (17.75)	141 (9.00)	< 0.0001
DM^b^					< 0.001				0.003				0.003
no	4050 (39.60)	1488 (37.64)	1677 (39.36)	885 (43.20)		1406 (41.03)	1363 (39.85)	1281 (37.64)		687 (36.81)	2858 (39.46)	505 (43.84)	
preDM	5111 (44.05)	1936 (43.74)	2163 (45.52)	1012 (41.73)		1769 (44.97)	1658 (43.37)	1684 (43.69)		897 (43.16)	3643 (44.62)	571 (41.91)	
DM	2499 (16.35)	1053 (18.61)	981 (15.13)	465 (15.08)		712 (14.01)	865 (16.77)	922 (18.66)		524 (20.03)	1738 (15.91)	237 (14.24)	
Energy intake, kcal/d^a^	2154.92 (11.18)	2125.59 (20.84)	2162.22 (20.07)	2187.68 (19.16)	0.13	2714.03 (20.07)	2097.93 (15.34)	1555.05 (14.00)	< 0.0001	1593.24 (20.43)	2218.87 (14.93)	2490.08 (29.49)	< 0.0001
Physical Activity, h/wk^a^	4664.67 (107.99)	5185.92 (166.61)	4610.22 (144.43)	4005.11 (157.81)	< 0.0001	4726.56 (143.58)	4610.03 (159.82)	4643.94 (210.80)	0.82	4633.75 (246.80)	4828.08 (126.61)	3860.30 (181.32)	< 0.0001

a : Weighted mean value (±standard deviation [SD])

b : Frequencies (proportions) as appropriate

Abbreviations: DI-GM, dietary index for gut microbiota; DII: dietary inflammation index; MASLD, metabolic dysfunction-associated steatotic liver disease; MetALD: metabolic dysfunction and alcohol-associated liver disease; Non-SLD, no steatotic liver disease; TyG, triglyceride- glucose index; MS, metabolic score; CRP, C-reactive protein; SII, systemic immune-inflammation index; SIRI, systemic inflammatory response index; preDM, prediabetes mellitus; DM: diabetes mellitus; CVD, cardiovascular diseases.

Furthermore, compared to healthy controls, patients with MASLD exhibited lower DI-GM scores and income levels, with the lowest values observed in MetALD patients (**Supplementary Table S1**). MASLD patients had a higher proportion of males, elevated DII scores, and increased levels of MS, CRP, SII, SIRI, daily energy intake, and physical activity, with the highest values noted in MetALD patients. MetALD patients also demonstrated higher TyG levels, and prevalence of CVD and diabetes, with the highest levels seen in MetALD patients. MASLD patients were the oldest, while MetALD patients were the youngest. Additionally, MASLD patients had the lowest proportion of smokers, whereas MetALD patients had the highest.

### 3.2 Association of DI-GM and DII with MASLD and MetALD

When analyzed as continuous variables, the DI-GM exhibited a significant inverse association with both MASLD (Crude model: β= 0.92, 95% CI = 0.89 to 0.95; Model 1: β= 0.90, 95% CI = 0.87 to 0.93; Model 2: β= 0.93, 95% CI = 0.89 to 0.97) and MetALD (Crude model: β= 0.87, 95% CI = 0.84 to 0.90; Model 1: β= 0.88, 95% CI = 0.84 to 0.91; Model 2: β= 0.91, 95% CI = 0.87 to 0.95). Conversely, the DII showed a significant positive association with the incidence of MASLD (Crude model: β= 1.07, 95% CI = 1.04 to 1.11; Model 1: β= 1.11, 95% CI = 1.07 to 1.14; Model 2: β= 1.09, 95% CI = 1.05 to 1.13) and MetALD (Crude model: β= 1.08, 95% CI = 1.04 to 1.12; Model 1: β= 1.11, 95% CI = 1.07 to 1.15; Model 2: β= 1.05, 95% CI = 1.05 to 1.1) (**Table 2**).

**Table 2:** Association of DI-GM, DII with MASLD and MetALD, weighted.

Variable	Crude model		Model 1		Model 2	
	β(95%CI)	p	β(95%CI)	p	β(95%CI)	p
MASLD						
DI-GM	0.92(0.89,0.95)	<0.0001	0.90(0.87,0.93)	<0.0001	0.93(0.89,0.97)	0.001
DII	1.07(1.04,1.11)	<0.0001	1.11(1.07,1.14)	<0.0001	1.09(1.05,1.13)	<0.0001
MetALD						
DI-GM	0.87(0.84,0.90)	<0.0001	0.88(0.84,0.91)	<0.0001	0.91(0.87,0.95)	<0.001
DII	1.08(1.04,1.12)	<0.0001	1.11(1.07,1.15)	<0.0001	1.05(1.00,1.10)	0.04

DI-GM, dietary index for gut microbiota; DII, dietary inflammation index; MASLD, metabolic dysfunction-associated steatotic liver disease; MetALD: metabolic dysfunction and alcohol-associated liver disease; β: Standardized Coefficients; CI: Confidence interval.

Crude model remained unadjusted;

Model 1 adjusted for age, sex;

Model 2 further adjusted for race/ethnicity, education level, marital status, PIR levels, diabetes, history of CVD, smoke, energy intake and physical activity.

**Figure 2:**
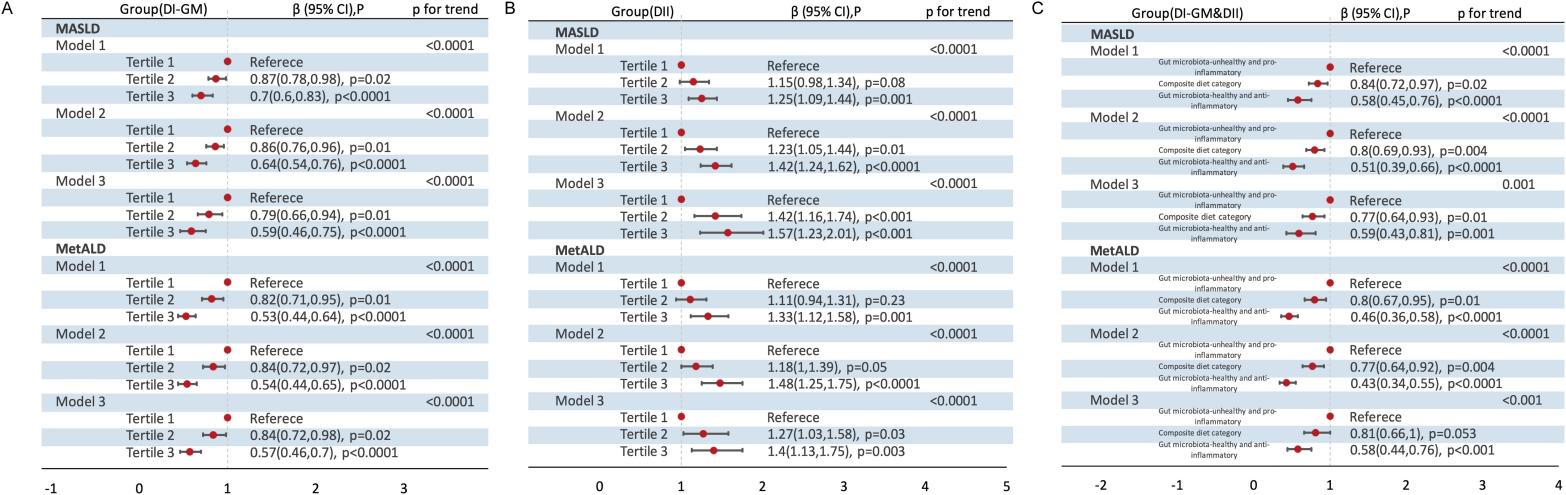
Association of DI-GM Tertiles, DII Tertiles and different combinations of DI-GM and DII with MASLD and MetALD, weighted. **(A)** DI-GM; **(B)** DII; **(C)** DI-GM&DII. DI-GM, dietary index for gut microbiota; DII, dietary inflammation index; MASLD, metabolic dysfunction-associated steatotic liver disease; MetALD, metabolic dysfunction and alcohol-associated liver disease; β, Standardized Coefficients; CI, Confidence interval. Crude model remained unadjusted; Model 1 adjusted for age, sex; Model 2 further adjusted for race/ethnicity, education level, marital status, PIR levels, diabetes, history of CVD, smoke, energy intake and physical activity. P for Trend: Tests for trends based on the variables containing the median values for each tertiles.

When DI-GM and DII were analyzed as categorical variables, a gut microbiota-healthy diet (DI-GM T3) and/or anti-inflammatory diet (DII T1) were significantly associated with a lower prevalence of MASLD and MetALD (**Figure 2**). Specifically, across the three models, compared to T1, DI-GM T3 was associated with a 41% reduction in MASLD prevalence (Crude model: β= 0.70, 95% CI = 0.60 to 0.83; Model 1: β= 0.64, 95% CI = 0.54 to 0.76; Model 2: β= 0.59, 95% CI = 0.46 to 0.75) and a 43% reduction in MetALD prevalence (Crude model: β= 0.53, 95% CI = 0.44 to 0.64; Model 1: β= 0.54, 95% CI = 0.44 to 0.65; Model 2: β= 0.57, 95% CI = 0.46 to 0.70). In contrast, DII Tertile 3 was significantly associated with an increased prevalence of MASLD (Crude model: β= 1.25, 95% CI = 1.09 to 1.44; Model 1: β= 1.42, 95% CI = 1.24 to 1.62; Model 2: β= 1.57, 95% CI = 1.23 to 2.01) and MetALD (Crude model: β= 1.33, 95% CI =1.12 to 1.58; Model 1: β= 1.48, 95% CI = 1.25 to 1.75; Model 2: β= 1.40, 95% CI =1.13 to 1.75). Furthermore, a combined gut microbiota-healthy and anti-inflammatory diet (DI-GM Tertile 3 and DII Tertile 1) was associated with a 41% reduction in MASLD prevalence (Crude model: β= 0.58, 95% CI =0.45 to 0.76; Model 1: β=0.51, 95% CI =0.39 to 0.66; Model 2: β= 0.59, 95% CI =0.43 to 0.81) and a 42% reduction in MetALD prevalence (Crude model: β= 0.46, 95% CI =0.36 to 0.58; Model 1: β=0.43, 95% CI =0.34 to 0.55; Model 2: β=0.58, 95% CI = 0.44 to 0.76) compared to a gut microbiota-healthy or anti-inflammatory diet alone.

### 3.3 Association of DI-GM Tertiles and DII Tertiles with MASLD and MetALD in MASLD and MetALD using the RCS

**Figure 3:**
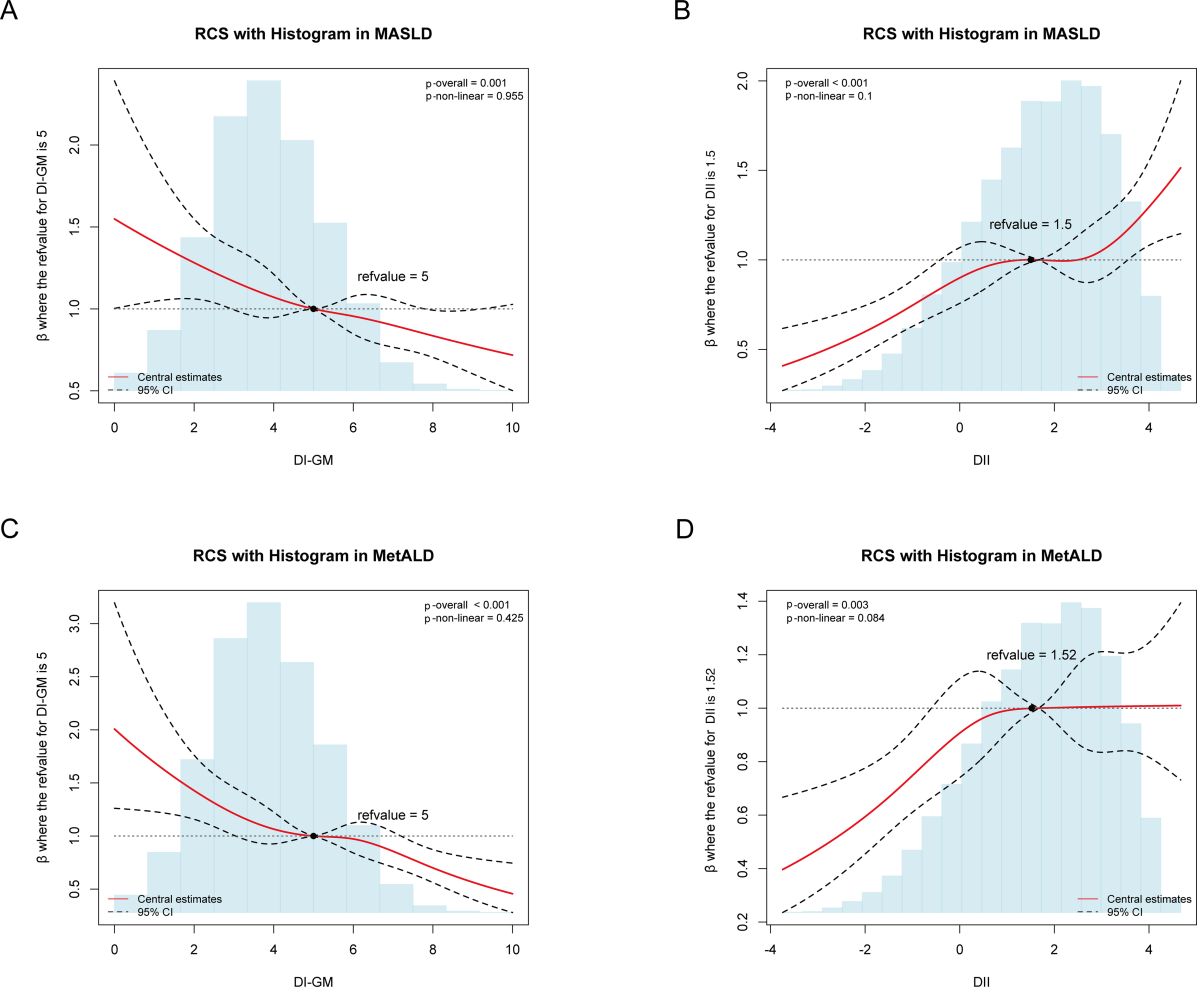
Association of DI-GM, DII with MASLD and MetALD using the RCS after adjustment for the covariables, weighted. **(A)** Association of DI-GM with MASLD. **(B)** Association of DII with MASLD. **(C)** Association of DI-GM with MetALD. DI-GM, dietary index for gut microbiota; DII, dietary inflammation index; MASLD, metabolic dysfunction-associated steatotic liver disease; MetALD, metabolic dysfunction and alcohol-associated liver disease; CI, Confidence interval.

Using RCS regression models to flexibly model the linear and dose-response relationships, both DI-GM and DII demonstrated approximately linear associations with MASLD (DI-GM: P_overall_ = 0.001, P_nonlinearity_ =0.955; DII: P_overall_ < 0.001, P_nonlinearity_ =0.1) and MetALD (DI-GM: P_overall_ < 0.001, P_nonlinearity_ =0.425; DII: P_overall_ =0.003, P_nonlinearity_ =0.084) after multivariable adjustment (**Figure 3**).

### 3.4 Association of DI-GM and DII with metabolic and inflammatory markers

**Figure 4:**
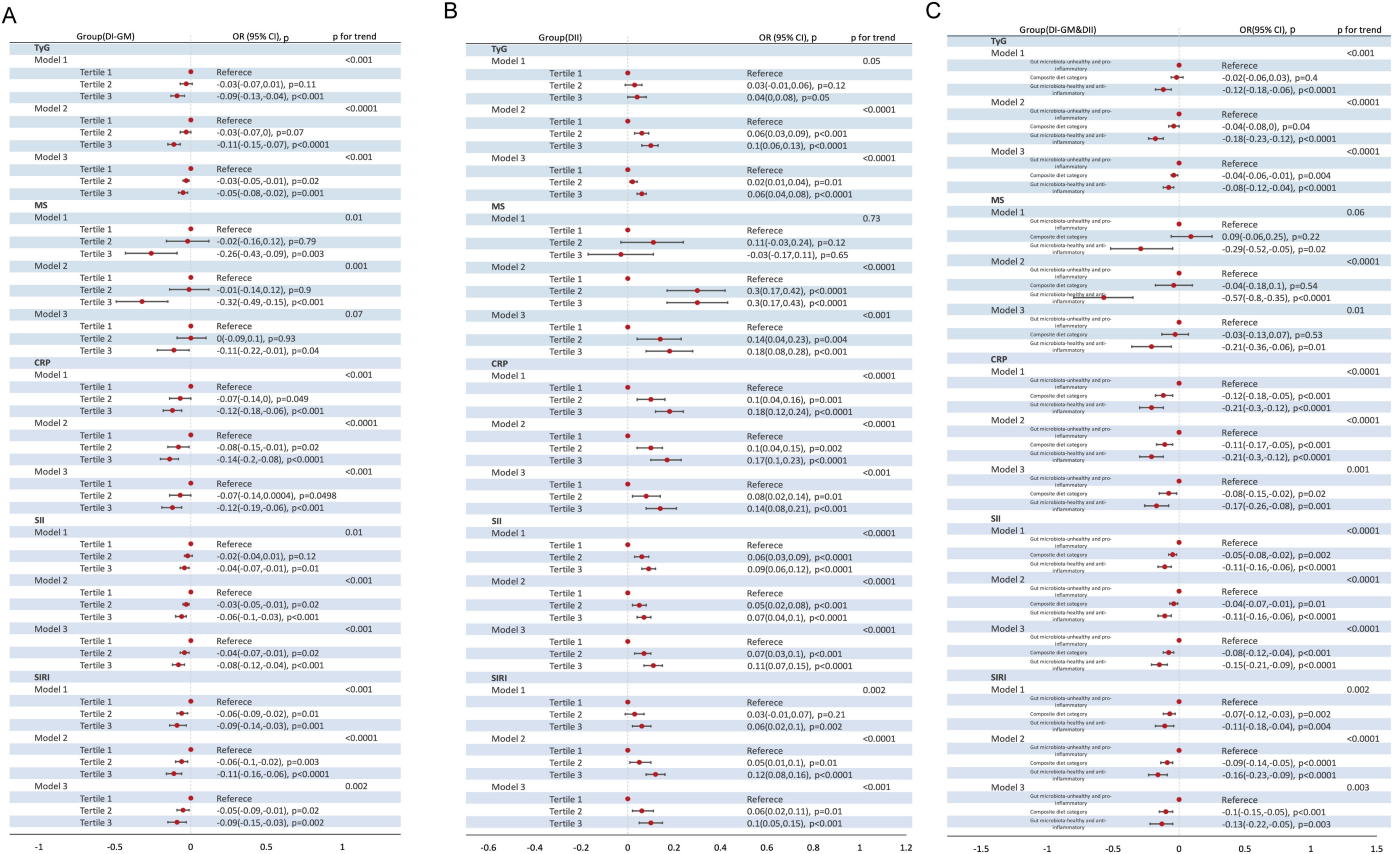
Association of DI-GM Tertiles, DII Tertiles and different combinations of DI-GM and DII with TyG, MS, CRP, SII, SIRI in NHANES 2007–2018 participants, weighted. **(A)** DI-GM; **(B)** DII; **(C)** DI-GM&DII. DI-GM, dietary index for gut microbiota; DII, dietary inflammation index; MASLD, metabolic dysfunction-associated steatotic liver disease; MetALD, metabolic dysfunction and alcohol-associated liver disease; TyG, triglyceride- glucose index; MS, metabolic score; CRP, C-reactive protein; SII, systemic immune-inflammation index; SIRI, systemic inflammatory response index; OR: odd ratio; CI: Confidence interval. Crude model remained unadjusted; Model 1 adjusted for age, sex; Model 2 further adjusted for race/ethnicity, education level, marital status, PIR levels, diabetes, history of CVD, smoke, energy intake and physical activity; P for Trend: Tests for trends based on the variables containing the median values for each tertiles.

In all three models, both the DI-GM and the DII were significantly associated with metabolic markers (TyG, MS) and inflammatory markers (CRP, SII, SIRI) (all P < 0.05) (**Table 3**, **Figure 4**), albeit in opposite directions. In Model 2, when DI-GM and DII were analyzed as continuous variables, DI-GM exhibited significant inverse associations with TyG, MS, CRP, SII, and SIRI (TyG: OR= -0.01, 95% CI = -0.01 to 0.00; MS: β= -0.02, 95% CI =-0.05 to 0.00; CRP: β= -0.02, 95% CI = -0.04 to -0.01; SII: β= -0.03, 95% CI = -0.04 to -0.01; SIRI: β= -0.03, 95% CI = -0.04 to -0.01). Conversely, DII showed significant positive associations with TyG, MS, CRP, SII, and SIRI (TyG: OR= 0.01, 95% CI = 0.01 to 0.02; MS: β=0.04, 95% CI =0.02 to 0.06; CRP: β= 0.03, 95% CI = 0.02 to 0.04; SII: β= 0.01, 95% CI = 0.01 to 0.02; SIRI: β= 0.02, 95% CI = 0.01 to 0.03). When DI-GM and DII were analyzed as categorical variables, these associations remained consistent (**Figure 4**).

**Table 3:** Association of DI-GM, DII with TyG, MS, CRP, SII, SIRI in NHANES 2007–2018 participants, weighted. .

Variable	Crude model		Model 1		Model 2	
	OR (95%CI)	p	OR (95%CI)	p	OR (95%CI)	p
TyG						
DI-GM	-0.02(-0.03, -0.01)	<0.0001	-0.02(-0.03, -0.01)	<0.0001	-0.01(-0.01, 0.00)	0.04
DII	0.01(0.00,0.02)	0.01	0.03(0.02,0.03)	<0.0001	0.01(0.01, 0.02)	<0.0001
MS						
DI-GM	-0.06(-0.10, -0.02)	0.002	-0.07(-0.10, -0.03)	<0.001	-0.02(-0.05, 0.00)	0.03
DII	0.01(-0.02,0.04)	0.53	0.09(0.06,0.12)	<0.0001	0.04(0.02, 0.06)	<0.0001
CRP						
DI-GM	-0.03(-0.04, -0.02)	<0.0001	-0.03(-0.05, -0.02)	<0.0001	-0.02(-0.04, -0.01)	0.003
DII	0.04(0.03,0.05)	<0.0001	0.04(0.03, 0.05)	<0.0001	0.03(0.02, 0.04)	<0.001
SII						
DI-GM	-0.03(-0.04, -0.02)	<0.0001	-0.03(-0.04, -0.02)	<0.0001	-0.03(-0.04, -0.01)	<0.0001
DII	0.02(0.01,0.03)	<0.0001	0.02(0.01, 0.02)	<0.0001	0.01(0.01, 0.02)	<0.0001
SIRI						
DI-GM	-0.03(-0.04, -0.02)	<0.0001	-0.03(-0.04, -0.02)	<0.0001	-0.03(-0.04, -0.01)	<0.0001
DII	0.02(0.01,0.02)	<0.001	0.03(0.02,0.04)	<0.0001	0.02(0.01, 0.03)	<0.0001

DI-GM, dietary index for gut microbiota; DII: dietary inflammation index; MASLD, metabolic dysfunction-associated steatotic liver disease; MetALD: metabolic dysfunction and alcohol-associated liver disease; TyG, triglyceride- glucose index; MS, metabolic score; CRP, C-reactive protein; SII, systemic immune-inflammation index; SIRI, Systemic inflammatory response index; OR: odd ratio; CI: Confidence interval.

Crude model remained unadjusted;

Model 1 adjusted for age, sex;

Model 2 further adjusted for race/ethnicity, education level, marital status, PIR levels, diabetes, history of CVD, smoke, energy intake and physical activity.

P for Trend: Tests for trends based on the variables containing the median values for each tertile.

### 3.5 Mediation analysis of dietary indices on MASLD/MetALD via metabolic and inflammatory markers

**Figure 5:**
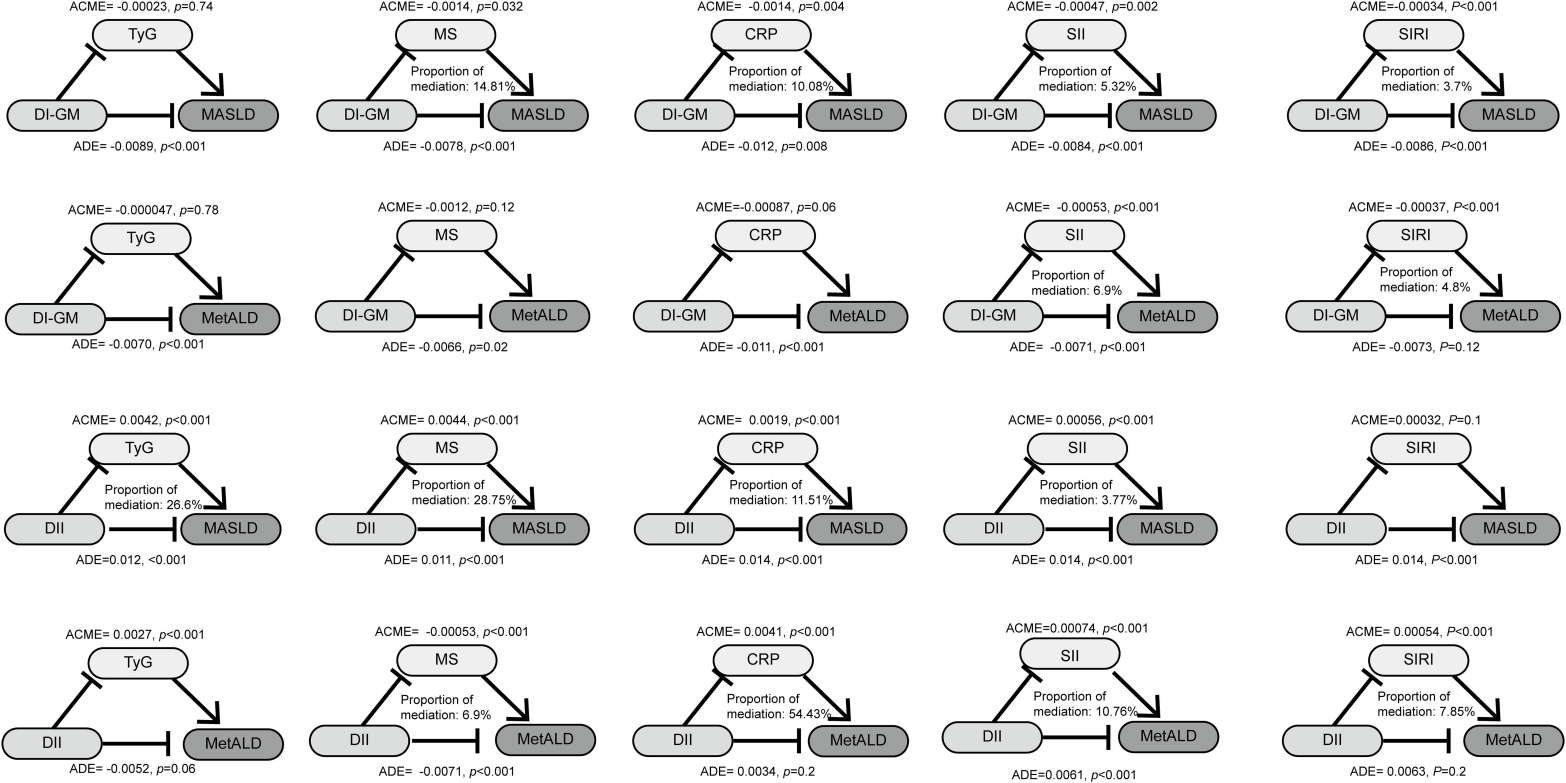
The mediated effects analysis of TyG, MS, CRP, SII, SIRI in the associations of DI-GM and DII with MASLD and MetALD. DI-GM, dietary index for gut microbiota; DII, dietary inflammation index; MASLD, metabolic dysfunction-associated steatotic liver disease; MetALD, metabolic dysfunction and alcohol-associated liver disease; TyG, triglyceride- glucose index; MS, metabolic score; CRP, C-reactive protein; SII, systemic immune-inflammation index; SIRI, systemic inflammatory response index; ACME, average causal mediation effects; ADE, average direct effects.

Given the associations between dietary indices, MASLD/MetALD, and the aforementioned metabolic and inflammatory markers, mediation analysis was employed to assess whether the effects of dietary indices on MASLD/MetALD are mediated by these markers (**Figure 5**). For the relationship between the DI-GM and MASLD, the indirect effects of MS, CRP, SII and SIRI accounted for 29.7%, 5.72%, 12.9%, and 25.4% of the total effect, respectively. Regarding the association between the DII and MASLD, the mediating effects of TyG, MS, CRP, and SII were 26.6%, 28.75%, 11.51%, and 3.77%, respectively. As for MetALD, DI-GM influenced its occurrence through the mediating effects of SII (6.9%) and SIRI (4.8%). Conversely, DII affected MetALD via the mediating roles of MS (6.9%), CRP (54.43%), SII (10.76%), and SIRI (7.85%).

### 3.6 Stratified analysis based on variables of interest

In extensive stratified analyses based on variables of interest, similar associations were observed (**Supplementary Table 2**-**7**). Notably, the results were strongly consistent with our primary findings (P<0.05) in subgroups of individuals aged <65 years, of non-Hispanic white, non-smokers, and without comorbidities such as diabetes or CVD.

## 4 Discussion

This cross-sectional study innovatively demonstrated that diet plays a role in reducing the incidence of MASLD/MetALD through the joint regulation of gut microbiota and systemic inflammation. Furthermore, the mediating roles of metabolic disorders and systemic inflammation in this relationship were investigated. A significant negative correlation was observed between DI-GM and the incidence of MASLD/MetALD, metabolic disorders, and systemic inflammatory responses, whereas DII exhibited an opposite trend. Notably, the combination of two beneficial dietary patterns resulted in a more pronounced reduction in the incidence of MASLD/MetALD, metabolic disorders, and inflammatory responses. Additionally, this beneficial effect was more pronounced and stable among non-Hispanic white individuals under 65 years of age who were non-smokers and had no history of diabetes or CVD complications. Ultimately, the reduction in metabolic disorders and inflammation was found to mediate the effect of gut microbiota-friendly and anti-inflammatory diets in lowering the incidence of MASLD and MetALD. While our findings highlight the association between dietary patterns and liver disease outcomes, the cross-sectional design of this study precludes causal inference. Reverse causality remains a potential confounder, as early-stage liver disease may alter dietary preferences or nutrient absorption, thereby influencing observed dietary patterns. Future cohort studies with repeated dietary assessments and long-term follow-up are urgently needed to clarify the temporal relationships and causal pathways between diet, gut microbiota, and liver disease progression.

Our study identifies a novel association between a gut microbiota-healthy (DI-GM T3) and/or anti-inflammatory diet (DII T1) with reduced risks of MASLD and MetALD. While Xumin et al. reported that anti-inflammatory diets lowered MASLD risk, they observed no incremental risk elevation with pro-inflammatory diets, suggesting a linear relationship (15). In contrast, Farhadnejad et al. demonstrated a positive association between continuous DII scores and MAFLD risk, though tertile-based comparisons revealed non-significant trends (16). Notably, Yan et al. described a U-shaped relationship between DII and MAFLD prevalence in a cross-sectional study of 3,633 participants (17). And Fanny Petermann-Rocha et al. recently identified nonlinear associations between pro-inflammatory diets and severe NAFLD risk in 171,544 UK Biobank participants (18). Diverging from these heterogeneous patterns, our results consistently demonstrated inverse linear associations of lower DII (both continuous and categorical) with reduced MASLD and MetALD risks. We believe the possible reasons are as follows: The transition from NAFLD to MAFLD and finally to MASLD reflects a shift toward emphasizing metabolic drivers over alcohol use. If earlier studies relied on older diagnostic criteria, this could lead to different associations. Additionally, our study population consisted of Americans, whereas the populations in Petermann-Rocha et al.’s studies were British. Geographic differences may also be a contributing factor to the discrepancies. Finally, the diagnostic approach for hepatic steatosis in our study differed from that used by Yan et al., which may also explain the variations. For DI-GM, our findings align with prior evidence from a 7,243-participant cross-sectional study showing a linear negative correlation between DI-GM and MAFLD prevalence (13). However, our work advances prior evidence by employing updated MASLD diagnostic criteria that rigorously integrate metabolic dysfunction parameters, thereby enhancing specificity and population generalizability. Our findings underscore the necessity of integrating gut microbiome preservation and anti-inflammatory nutrient optimization into dietary strategies for MASLD/MetALD prevention. Future research should validate these associations longitudinally and explore thresholds for therapeutic interventions.

In exploring the underlying mechanisms linking these assciations, we identified critical interactions between gut microbiota, systemic inflammation, and liver pathology in the pathogenesis of MASLD and MetALD. Research has demonstrated that MASLD patients experience intestinal bacterial overgrowth and significant alterations in gut microbiota composition (43–45). The altered gut microbiota can directly disrupt the intestinal epithelium and vascular barrier (46). At the same time, subclinical intestinal inflammation occurs, which is characterized by a decrease in lamina propria regulatory T (Treg) cells, an increase in T helper 1 (Th1) and cluster of differentiation 8 positive (CD8+) T cells that produce interferon - γ (IFN - γ), and an increase in gamma - delta (γδ) T cells that produce interleukin - 17 (IL - 17) (47). Additionally, MASLD patients exhibit reduced expression of the intestinal epithelial junctional adhesion molecule Jam1 in the colon, leading to colonic inflammation (48). Preclinical studies show that junctional adhesion molecule 1 (Jam1)-deficient mice fed high-fat, high-fructose diets develop increased intestinal permeability, endotoxemia, and liver inflammation (48). Previous studies have shown that dietary patterns are one of the important factors that can alter the gut microbiota in humans (49, 50). For instance, western-style diets (high in fat, cholesterol, and refined carbohydrates) induce microbial dysbiosis, favoring pro-inflammatory bacterial communities (e.g., *Proteobacteria*) and reducing protective commensals (e.g., Bacteroidetes/Firmicutes), ultimately exacerbating systemic inflammation and metabolic dysfunction (51, 52). These dietary effects may enhance intestinal permeability, facilitating translocation of pathogen-associated molecular patterns (PAMPs), such as lipopolysaccharide (LPS) and microbiota-derived metabolites, thereby triggering pro-inflammatory cascades and worsening non-hepatic inflammation and metabolic aberrations, particularly in the presence of subclinical pathology (e.g., hepatocellular lipid accumulation) (53, 54). Conversely, microbial fermentation of undigestible carbohydrates (e.g., dietary fiber) generates short-chain fatty acids (SCFAs), which serve as energy substrates for colonic epithelia, attenuate gut inflammation, and modulate satiety, thereby improving MASLD outcomes (55, 56). Additionally, they can also function as prebiotics by promoting the proliferation of beneficial bacteria like Bifidobacterium and Lactobacillus, thereby enhancing the synthesis of short-chain fatty acids (SCFAs) such as butyrate and propionate (19, 57). These SCFAs activate intestinal endocrine cells via G protein-coupled receptors (GPR41/GPR43), stimulating the secretion of gut hormones (e.g., GLP-1, PYY) to regulate insulin sensitivity and inhibit hepatic lipid synthesis (58). Meanwhile, SCFAs enhance intestinal barrier function by inhibiting histone deacetylases (HDACs), reducing gut permeability and preventing bacterial lipopolysaccharides (LPS) from entering the circulation, thus attenuating systemic inflammation mediated by the TLR4 signaling pathway (59). Additionally, probiotics in fermented dairy products (e.g., *Firmicutes*) further promote SCFA production, establishing a positive feedback loop in “gut-liver axis signaling” (60). In contrast, the pro-inflammatory components of DII (e.g., refined carbohydrates) promote gut microbiota dysbiosis (e.g., increased Bacteroidetes abundance and imbalanced Firmicutes/Bacteroidetes ratio), leading to elevated propionate production among short-chain fatty acids (SCFAs) (61). Propionate may induce the release of inflammatory cytokines such as IL-6 and TNF-α by activating the NF-κB pathway (62). The anti-inflammatory effects of n-3 polyunsaturated fatty acids (e.g., eicosapentaenoic acid [EPA] and docosahexaenoic acid [DHA]) are attributed to their ability to generate specific lipid mediators that modulate inflammatory responses (63). Inflammatory processes are characterized by increased levels of cytokines such as prostaglandins, IL-6, IL-2, TNF-α, C-reactive protein (CRP), and reactive oxygen species (ROS) (63, 64). For MetALD patients, alcohol intake directly alters gut microbial composition, as evidenced by reduced bacterial diversity, decreased *Bacteroidetes/Firmicutes*, and increased *Proteobacteria* enrichment in alcohol-fed mice (65, 66). This dysbiosis also reduces fungal diversity and promotes *Candida* overgrowth, with subsequent systemic immunogenic responses triggered by fungal β-glucans (67). Concurrently, alcohol damages gut barrier components, such as regenerating islet-derived 3-β (Reg3b) and 3-γ (Reg3g) proteins involved in innate antimicrobial defense, leading to increased bacterial adhesion, excessive gut bacterial overgrowth, translocation of viable bacteria, and exacerbated liver inflammation (68, 69). Alcohol-induced gut dysbiosis reduces the microbiota’s capacity to synthesize saturated long-chain fatty acids (LCFA), thereby decreasing the proportion of LCFA-dependent microbial populations, such as *Lactobacillus* species (65). However, dietary supplementation with saturated LCFA can restore gut ecology, stabilize gut barriers, and attenuate ethanol-induced hepatotoxicity (65). Additionally, alcohol-induced microbial dysbiosis alters bile acid homeostasis, increasing gut bile acid deconjugation and exposing hepatocytes to more toxic bile acid species (70). These findings underscore the therapeutic potential of modulating gut microbiota and attenuating systemic inflammation in the prevention and management of MASLD and MetALD.

Our study revealed another significant finding: a synergistic effect of a healthy gut microbiome diet (DI-GM Tertile 3) and an anti-inflammatory diet (DII Tertile 1) in reducing the risk of MASLD and MetALD. We hypothesize that this effect arises from the interaction between the two diets. Specifically, the gut microbiome plays a pivotal role in regulating pro-inflammatory and anti-inflammatory responses within the intestine (71). Mechanically, dysbiosis of the gut microbiota can compromise intestinal barrier integrity, increasing gut permeability (the “leaky gut” phenomenon), which allows bacterial metabolites (e.g., LPS) and PAMPs to translocate into the portal circulation (72). In the liver, LPS activates immune cells such as Kupffer cells through the toll-like receptor 4 (TLR4) signaling pathway, triggering systemic inflammation and the production of pro-inflammatory cytokines, including tumor necrosis factor-α (TNF-α), interleukin-6 (IL-6), and interleukin-1β (IL-1β) (72). Concurrently, gut microbiota dysbiosis reduces the production of SCFAs, which are anti-inflammatory mediators, and this imbalance leads to increased pro-inflammatory cytokines and decreased anti-inflammatory cytokines (73). Moreover, dysbiosis also alters bile acid profiles, impairing the activity of the farnesoid X receptor (FXR) and the G protein-coupled bile acid receptor 1 (TGR5), which diminishes their anti-inflammatory and metabolic regulatory roles (73). Additionally, dysbiosis promotes the activation of pro-inflammatory immune cells, such as M1 macrophages, which release excessive pro-inflammatory cytokines, exacerbating liver inflammation and injury (74, 75). Furthermore, gut microbiota dysbiosis depletes endogenous antioxidants, such as glutathione, and increases reactive oxygen species (ROS) generation (76). ROS impair mitochondrial function in hepatocytes, disrupting energy metabolism and amplifying inflammatory responses (77). On the other hand, anti-inflammatory diets promote the growth of beneficial microbial species, such as *Bifidobacterium* and *Lactobacillus*, while inhibiting the proliferation of harmful bacteria, such as *Prevotella copri (* 78–80). On the other hand, SCFAs (e.g., propionate) promoted by DI-GM enhance insulin sensitivity by activating the AMPK pathway (81), while the low-inflammatory environment of DII reduces the interference of inflammatory factors on insulin signaling, jointly improving metabolic disorders (18). In conclusion, the synergistic effects of a healthy gut microbiome and an anti-inflammatory diet may surpass the individual benefits of either diet alone, suggesting a greater-than-additive protective effect against MASLD and MetALD.

We further observed that TyG, MS, CRP, SII, and SIRI mediated the correlation between a healthy gut microbiota (DIGM T3) and/or anti-inflammatory diet (DII T1) and the reduced prevalence of MASLD/MetALD, highlighting their potential health benefits. TyG and MS, as biomarkers of metabolic dysfunction, our findings and previous studies suggest that elevated DII exacerbates unhealthy metabolic states (27, 28, 82). A higher DII is associated with increased impaired glucose homeostasis and lipid particle numbers, including low-density lipoprotein (VLDL), low-density lipoprotein (LDL), and high-density lipoprotein (HDL) (83). For DI-GM, although its relationship with metabolic status and inflammation remains understudied, its mechanisms are likely linked to the modulation of host metabolic processes by the gut microbiota, including energy balance, glucose metabolism, and lipid metabolism (84). Additionally, a 17-week randomized prospective study demonstrated that diets promoting a healthy gut microbiome (e.g., high-fiber or fermented diets) can regulate immune function and reduce systemic inflammation (85).

Systemic metabolism and inflammation contribute significantly to the pathogenesis of MASLD and MetALD. Mechanically, inflammation disrupts fat tissue function, increasing the release of free fatty acids (FFAs), which accumulate in the liver and contribute to steatosis (86). Inflammation also alters adipocytokine secretion patterns, promoting pro-inflammatory factors (e.g., leptin) and reducing anti-inflammatory factors (e.g., adiponectin), and this imbalance enhances liver fat synthesis and accumulation (87). Furthermore, inflammatory cytokines (e.g., TNF-α and IL-6) impair insulin signaling, leading to insulin resistance, which stimulates liver fat synthesis and inhibits fat degradation, exacerbating liver steatosis (88). Additionally, inflammatory responses increase reactive oxygen species (ROS), which causes oxidative stress, and ROS induce lipid peroxidation, producing cytotoxic lipids that damage hepatocytes and perpetuate inflammation (77). As for metabolism disorders, dysregulated glucose metabolism and insulin resistance stimulate insulin secretion, increasing hepatic triglyceride synthesis, which elevates plasma triglycerides and promotes NAFLD progression (89). Similarly, lipid metabolism abnormalities, in which excess triglycerides arise from hepatic *de novo* lipogenesis and dietary fat intake while FFAs result from visceral fat tissue lipolysis, are crucial in NAFLD development (90). FFAs contribute to MASLD via mitochondrial β-oxidation, endoplasmic reticulum stress, lysosomal dysfunction, and cell death, and furthermore, free cholesterol (FC), which is recognized as a hepatotoxic agent, also plays a role (91). Our findings reinforce the association between dietary inflammation and gut microbiota modulation with MASLD, which suggest that reducing systemic inflammation and regulating gut microbiota through a gut microbiota-healthy (DI-GM T3) and/or anti-inflammatory diet (DII T1) may help mitigate MASLD progression. Nonetheless, additional mechanisms linking dietary inflammation and gut microbiota modulation warrant further exploration in future studies.

Our findings demonstrated that the beneficial effects of a gut microbiota-healthy (DI-GM T3) and/or anti-inflammatory diet (DII T1) on MASLD/MetALD were more pronounced and consistent among specific subgroups, namely individuals younger than 65 years, non-smokers, and obese individuals without diabetes or CVD comorbidities. This observation suggests that the protective role of these dietary factors may be particularly robust in populations with fewer confounding health conditions or modifiable risk factors. From a mechanistic perspective, the higher levels of gut microbiota dysbiosis and systemic inflammation observed in older adults, smokers, and individuals with diabetes or CVD-related complications likely contribute to the attenuation of the beneficial effects of DI-GM and DII on MASLD/MetALD progression (92–95). These conditions are often associated with chronic low-grade inflammation and impaired gut integrity, which may reduce the capacity of a healthy gut microbiota and anti-inflammatory diet to exert their protective effects. This observation was particularly evident among non-Hispanic white participants, where the beneficial associations aligned closely with our preliminary findings and corroborated findings from Zheng et al.’s investigation (13). The racial homogeneity in gut microbiota modulation may be attributed to ethnicity-specific influences on microbial composition that extend beyond geographic determinants to encompass distinct lifestyle patterns and cultural practices (96–98).

This study has several strengths. First, to the best of our knowledge, this is the first study to investigate the relationship between different combinations of DII and DI-GM with MASLD, MetALD, and metabolic dysregulation, as well as inflammation markers using a large-scale database. Combining these two dietary indices provides more precise dietary recommendations for the prevention of MASLD. Second, the utilization of a sophisticated, multi-stage probability sampling method ensures that the participants in this study represent the civilian non-institutionalized population, enabling generalizability of the findings across the United States. Third, compared to other dietary indices, the inclusion of fermented dairy in the DI-GM provides additional healthy dietary options for the prevention of MASLD and MetALD (8). Fourth, for the first time, we have assessed the roles of metabolic dysregulation markers (TyG, MS) and inflammation markers (CRP, SII, SIRI) as potential mediators in reducing the incidence of MASLD and MetALD attributed to a gut microbiota-healthy (DI-GM T3) and/or an anti-inflammatory diet (DII T1).

Despite these strengths, our study has some limitations. First, the assessment of dietary intake, including DI-GM and DII, relied on the average of two 24-hour dietary recall interviews, which is prone to recall bias and only short-term dietary fluctuations, while failing to reflect the long-term dietary exposure required for chronic disease development. Additionally, the diagnoses of CVD, diabetes, smoking status, cancer, MASLD, and MetALD were self-reported, potentially introducing reporting bias. Second, selection bias may have occurred due to the exclusion of numerous participants with missing data or incomplete records. Third, as a cross-sectional study, we were unable to establish causality between dietary factors and MASLD/MetALD. Fourth, while patients were categorized into MASLD and MetALD groups based on their baseline alcohol consumption, prospective changes in drinking habits were not considered. Therefore, future prospective studies should account for variations in alcohol consumption or SLD progression. Fifth, alcohol use was assessed via self-reported data, which may introduce bias. However, self-reported alcohol use has been demonstrated to be a reliable and valid method for assessing alcohol consumption (99). Sixth, this study employed FLI ≥60 as a non-invasive diagnostic criterion for SLD, which may offer practical advantages in primary MASLD case selection compared to histological or ultrasound-based assessments (100). However, it should be noted that FLI demonstrates limited diagnostic accuracy in differentiating between distinct etiologies of SLD. Seventh, this study is the reliance on NHANES data from 2007–2018. While recent dietary trends may differ due to evolving food environments, post-2018 data became unusable for DIGM/DII calculations due to incomplete of standardized food composition reporting. Future studies should prioritize longitudinal datasets with updated DI-GM and DII metrics to address this gap. Lastly,

## 5 Conclusion

In summary, a gut microbiota-healthy (DI-GM T3) and anti-inflammatory (DII T1) diet synergistically mitigate the incidence of MASLD and MetALD through multifaceted mechanisms, including inflammation reduction, metabolic health improvement. These findings underscore the potential of dietary interventions targeting inflammation and gut microbiome modulation as novel therapeutic avenues for the prevention and management of metabolic liver diseases.

## Data Availability

The original contributions presented in the study are included in the article/supplementary material. Further inquiries can be directed to the corresponding author.
