# Supplementary material for: Association of the dietary index for gut microbiota and dietary inflammation index with metabolic dysfunction-associated steatotic liver disease and metabolic alcohol-associated liver disease
Source: Front Immunol. 2025 Jul 17;16:1593245. doi: 10.3389/fimmu.2025.1593245 (PMC12310490; doi:10.3389/fimmu.2025.1593245)
Supplement: Supplementary file 1 [file Table1.docx]

Supplementary Material

# Supplementary Tables

Table S1 Weighted sample characteristics of Non-SLD, MASLD and MetALD paiticipants in NHANES 2007–2018.

|  | **Non-SLD** | **MASLD** | **MetALD** | **p-value** |
| --- | --- | --- | --- | --- |
| **DI-GM^a^** | 5.34(0.04) | 5.07(0.04) | 4.85(0.05) | < 0.0001 |
| **DII^a^** | 1.28(0.04) | 1.53(0.04) | 1.63(0.05) | < 0.0001 |
| **TyG^a^** | 8.29(0.01) | 8.99(0.02) | 8.93(0.02) | < 0.0001 |
| **MS^a^** | -1.11(0.04) | 1.13(0.06) | 1.21(0.07) | < 0.0001 |
| **CRP^a^** | 0.26(0.01) | 0.527(0.03) | 0.531(0.02) | < 0.0001 |
| **SII^a^** | 6.06(0.01) | 6.17(0.01) | 6.19(0.01) | < 0.0001 |
| **SIRI^a^** | 1.10(0.02) | 1.26(0.02) | 1.29(0.02) | < 0.0001 |
| **Age^a^** | 46.84(0.37) | 53.29(0.37) | 46.53(0.45) | < 0.0001 |
| **Male^b^** | 2906(43.22) | 1613(52.85) | 1020(52.98) | < 0.0001 |
| **Race and ethnicity^b^** |  |  |  | < 0.0001 |
| Mexican American | 822( 6.88) | 510( 7.88) | 402(12.32) |  |
| Non-Hispanic Black | 1261( 9.77) | 656(10.14) | 406(11.09) |  |
| Non-Hispanic White | 2825(69.14) | 1444(71.04) | 807(66.37) |  |
| Other Race | 1620(14.21) | 572(10.94) | 335(10.22) |  |
| **Less than high school^b^** | 1093(11.45) | 664(15.04) | 403(14.38) | < 0.001 |
| **Married^b^** | 3840(61.20) | 2142(70.42) | 1034(55.15) | < 0.0001 |
| **Poverty income ratio level^b^** |  |  |  | < 0.0001 |
| High | 1990(45.69) | 831(41.01) | 447(36.72) |  |
| Low | 1725(19.53) | 916(20.12) | 641(24.90) |  |
| Middle | 2262(34.78) | 1145(38.87) | 706(38.38) |  |
| **CVD^b^** | 565( 6.96) | 508(13.98) | 264( 9.71) | < 0.0001 |
| **History of Cancer^b^** | 583( 9.43) | 378(12.31) | 161( 8.98) | 0.01 |
| **Smoke^b^** | 1248(18.27) | 403(11.04) | 552(27.49) | < 0.0001 |
| **DM^b^** |  |  |  | < 0.0001 |
| no | 3024(51.77) | 576(21.45) | 450(26.75) |  |
| preDM | 2722(40.36) | 1461(48.26) | 928(50.01) |  |
| DM | 782( 7.87) | 1145(30.29) | 572(23.24) |  |
| **Energy intake, kcal/d^a^** | 2127.85(15.52) | 2143.25(23.93) | 2267.16(34.41) | 0.003 |
| **Physical Activity, h/wk^a^** | 4815.84(128.15) | 4277.09(184.41) | 4697.34(197.51) | 0.03 |

^a^: Weighted mean value (±standard deviation [SD])

^b^: Frequencies (proportions) as appropriate

Abbreviations: DI-GM, dietary index for gut microbiota; DII: dietary inflammation index; MASLD, metabolic dysfunction-associated steatotic liver disease; MetALD: metabolic dysfunction and alcohol-associated liver disease; Non-SLD, no steatotic liver disease; TyG, triglyceride- glucose index; MS, metabolic score; CRP, C-reactive protein; SII, systemic immune-inflammation index; SIRI, Systemic inflammatory response index; preDM, prediabetes mellitus; DM: diabetes mellitus; CVD, cardiovascular diseases.

**Table S2** **Association of DI-GM Tertiles with MASLD stratified by variables of interest.**

|  | **DI-GM** | | | | | |
| --- | --- | --- | --- | --- | --- | --- |
|  | **T1** | **T2** | | **T3** | |  |
|  |  | **β (95%CI)** | **p** | **β (95%CI)** | **p** | **p for trend** |
| **Sex** |  |  |  |  |  |  |
| Male | Ref. | 0.84(0.65,1.09) | 0.19 | 0.64(0.47,0.88) | 0.01 | 0.01 |
| Female | Ref. | 0.81(0.62,1.04) | 0.10 | 0.59(0.43,0.82) | 0.002 | 0.002 |
| **Age** | Ref. |  |  |  |  |  |
| < 65 yrs | Ref. | 0.85(0.73,1.01) | 0.06 | 0.65(0.52,0.81) | <0.001 | <0.001 |
| >= 65 yrs | Ref. | 0.78(0.58,1.06) | 0.12 | 0.68(0.48,0.96) | 0.03 | 0.03 |
| **Race** |  |  |  |  |  |  |
| Mexican American | Ref. | 0.82(0.59,1.13) | 0.22 | 0.93(0.61,1.41) | 0.72 | 0.46 |
| Other Race | Ref. | 0.77(0.58,1.02) | 0.07 | 0.60(0.41,0.89) | 0.01 | 0.01 |
| Non-Hispanic White | Ref. | 0.90(0.72,1.13) | 0.36 | 0.65(0.49,0.85) | 0.003 | 0.004 |
| Non-Hispanic Black | Ref. | 0.82(0.64,1.04) | 0.10 | 1.24(0.89,1.71) | 0.20 | 0.71 |
| **Smoke** |  |  |  |  |  |  |
| No | Ref. | 0.83(0.70, 0.99) | 0.04 | 0.57(0.46, 0.70) | <0.0001 | <0.0001 |
| Yes | Ref. | 0.77(0.56, 1.06) | 0.11 | 0.73(0.39, 1.39) | 0.34 | 0.17 |
| **CVD** |  |  |  |  |  |  |
| No | Ref. | 0.84(0.71, 0.99) | 0.04 | 0.58(0.47, 0.72) | <0.0001 | <0.0001 |
| Yes | Ref. | 0.81(0.54, 1.20) | 0.28 | 0.55(0.29, 1.03) | 0.06 | 0.05 |
| **preDM/DM** |  |  |  |  |  |  |
| No | Ref. | 0.95(0.72,1.24) | 0.68 | 0.50(0.34,0.74) | <0.001 | <0.001 |
| preDM | Ref. | 0.68(0.56,0.82) | <0.001 | 0.53(0.40,0.70) | <0.0001 | <0.0001 |
| DM | Ref. | 1.19(0.83,1.70) | 0.33 | 0.92(0.57,1.47) | 0.72 | 0.89 |

Each subgroup adjusted for all factors in model 2 except the stratification factor itself.

Abbreviations: DI-GM, dietary index for gut microbiota; T, tertile; MASLD, metabolic dysfunction-associated steatotic liver disease; preDM, prediabetes mellitus; DM: diabetes mellitus; CVD, cardiovascular diseases.

β: Standardized Coefficients; CI: Confidence interval.

P for Trend: Tests for trends based on the variables containing the median values for each tertile.

Table S3 Association of DII Tertiles with MASLD stratified by variables of interest.

|  | **DII** | | | | | |
| --- | --- | --- | --- | --- | --- | --- |
|  | **T1** | **T2** | | **T3** | |  |
|  |  | **β (95%CI)** | **p** | **β (95%CI)** | **p** | **p for trend** |
| **Sex** |  |  |  |  |  |  |
| Male | Ref. | 1.33(1.06, 1.67) | 0.01 | 1.33(1.04, 1.70) | 0.02 | 0.02 |
| Female | Ref. | 1.15(0.74, 1.78) | 0.53 | 1.59(1.03, 2.47) | 0.04 | 0.04 |
| **Age** | Ref. |  |  |  |  |  |
| < 65 yrs | Ref. | 1.23(0.13,11.23) | 0.86 | 4.90(1.13,21.23) | 0.03 | 0.03 |
| >= 65 yrs | Ref. | 1.24(1.02,1.52) | 0.04 | 1.35(1.08,1.67) | 0.01 | 0.01 |
| **Race** |  |  |  |  |  |  |
| Mexican American | Ref. | 1.02(0.61, 1.70) | 0.94 | 1.10(0.63, 1.93) | 0.73 | 0.75 |
| Other Race | Ref. | 1.41(0.87, 2.29) | 0.16 | 1.60(0.90, 2.84) | 0.11 | 0.1 |
| Non-Hispanic White | Ref. | 1.31(1.01, 1.71) | 0.04 | 1.41(1.06, 1.88) | 0.02 | 0.02 |
| Non-Hispanic Black | Ref. | 1.09(0.76, 1.56) | 0.62 | 1.14(0.75, 1.75) | 0.54 | 0.55 |
| **Smoke** |  |  |  |  |  |  |
| No | Ref. | 1.27(1.04,1.55) | 0.02 | 1.47(1.17,1.85) | 0.001 | 0.001 |
| Yes | Ref. | 1.62(1.01, 2.62) | 0.05 | 1.80(1.06, 3.06) | 0.03 | 0.04 |
| **CVD** |  |  |  |  |  |  |
| No | Ref. | 1.32(1.08,1.61) | 0.01 | 1.50(1.21,1.87) | <0.001 | <0.001 |
| Yes | Ref. | 1.09(0.61, 1.95) | 0.77 | 1.64(0.89, 3.02) | 0.11 | 0.11 |
| **preDM/DM** |  |  |  |  |  |  |
| No | Ref. | 1.38(0.92,2.06) | 0.12 | 1.71(1.13,2.59) | 0.01 | 0.01 |
| preDM | Ref. | 1.30(1.01,1.66) | 0.04 | 1.44(1.05,1.96) | 0.02 | 0.02 |
| DM | Ref. | 1.07(0.74,1.54) | 0.72 | 1.35(0.92,1.98) | 0.12 | 0.12 |

Each subgroup adjusted for all factors in model 2 except the stratification factor itself.

Abbreviations: DII: dietary inflammation index; T, tertile; MASLD, metabolic dysfunction-associated steatotic liver disease; preDM, prediabetes mellitus; DM: diabetes mellitus; CVD, cardiovascular diseases.

β: Standardized Coefficients; CI: Confidence interval.

P for Trend: Tests for trends based on the variables containing the median values for each tertile.

Table S4 Association of different combinations of DI-GM and DII with MASLD stratified by variables of interest.

|  | **DII&DIGM** | | | | | |
| --- | --- | --- | --- | --- | --- | --- |
|  | **Gut microbiota-unhealthy and pro-inflammatory** | **Composite diet category** | | **Gut microbiota-healthy and anti-inflammatory** | |  |
|  |  | **β (95%CI)** | **p** | **β (95%CI)** | **p** | **p for trend** |
| **Sex** |  |  |  |  |  |  |
| Male | Ref. | 1.74(1.25,2.44) | 0.001 | 2.32(1.52,3.54) | <0.001 | <0.0001 |
| Female | Ref. | 1.60(1.13, 2.25) | 0.01 | 1.86(1.26, 2.74) | 0.002 | 0.003 |
| **Age** |  |  |  |  |  |  |
| < 65 yrs | Ref. | 1.60(1.22,2.10) | <0.001 | 1.96(1.41,2.72) | <0.001 | <0.0001 |
| >= 65 yrs | Ref. | 1.07(0.72,1.60) | 0.73 | 1.67(1.03,2.71) | 0.04 | 0.03 |
| **Race** |  |  |  |  |  |  |
| Mexican American | Ref. | 1.35(0.61, 3.01) | 0.45 | 1.73(0.70, 4.29) | 0.23 | 0.21 |
| Other Race | Ref. | 1.58(0.88, 2.84) | 0.12 | 1.51(0.62, 3.70) | 0.36 | 0.34 |
| Non-Hispanic White | Ref. | 1.46(1.03, 2.06) | 0.03 | 1.74(1.15, 2.64) | 0.01 | 0.01 |
| Non-Hispanic Black | Ref. | 0.91(0.52, 1.60) | 0.74 | 0.95(0.49, .87) | 0.89 | 0.98 |
| **Smoke** |  |  |  |  |  |  |
| No | Ref. | 1.70(1.33,2.17) | <0.0001 | 2.04(1.44,2.87) | <0.0001 | <0.0001 |
| Yes | Ref. | 1.07(0.49, 2.34) | 0.87 | 1.50(0.62, 3.59) | 0.36 | 0.18 |
| **CVD** |  |  |  |  |  |  |
| No | Ref. | 1.63(1.32,2.01) | <0.0001 | 2.03(1.52,2.71) | <0.0001 | <0.0001 |
| Yes | Ref. | 0.69(0.35, 1.32) | 0.26 | 0.91(0.42, 1.98) | 0.82 | 0.86 |
| **preDM/DM** |  |  |  |  |  |  |
| No | Ref. | 2.40(1.51,3.82) | <0.001 | 2.64(1.44,4.85) | 0.002 | <0.001 |
| preDM | Ref. | 1.47(1.09,1.97) | 0.01 | 2.12(1.43,3.15) | <0.001 | <0.001 |
| DM | Ref. | 1.26(0.72,2.19) | 0.41 | 1.34(0.69,2.61) | 0.38 | 0.41 |

Each subgroup adjusted for all factors in model 2 except the stratification factor itself.

Abbreviations: DI-GM, dietary index for gut microbiota; DII: dietary inflammation index; MASLD, metabolic dysfunction-associated steatotic liver disease; preDM, prediabetes mellitus; DM: diabetes mellitus; CVD, cardiovascular diseases.

β: Standardized Coefficients; CI: Confidence interval.

P for Trend: Tests for trends based on the variables containing the median values for each tertile.

Table S5 Association of DI-GM Tertiles with MetALD stratified by variables of interest.

|  | **DI-DM** | | | | | |
| --- | --- | --- | --- | --- | --- | --- |
|  | **T1** | **T2** | | **T3** | |  |
|  |  | **β (95%CI)** | **p** | **β (95%CI)** | **p** | **p for trend** |
| **Sex** |  |  |  |  |  |  |
| Male | Ref. | 0.74(0.61,0.90) | 0.004 | 0.49(0.34,0.71) | <0.001 | <0.0001 |
| Female | Ref. | 0.97(0.74,1.27) | 0.84 | 0.56(0.39,0.80) | 0.002 | 0.003 |
| **Age** |  |  |  |  |  |  |
| < 65 yrs | Ref. | 0.79(0.67,0.93) | 0.01 | 0.49(0.36,0.66) | <0.0001 | <0.0001 |
| >= 65 yrs | Ref. | 1.38(0.76,2.51) | 0.29 | 0.79(0.36,1.71) | 0.54 | 0.56 |
| **Race** |  |  |  |  |  |  |
| Mexican American | Ref. | 0.95(0.68,1.33) | 0.78 | 0.54(0.31,0.94) | 0.03 | 0.05 |
| Other Race | Ref. | 0.64(0.44, 0.91) | 0.01 | 0.30(0.18, 0.49) | <0.0001 | <0.0001 |
| Non-Hispanic White | Ref. | 0.85(0.68,1.06) | 0.14 | 0.58(0.40,0.82) | 0.003 | 0.002 |
| Non-Hispanic Black | Ref. | 0.91(0.65,1.28) | 0.59 | 0.81(0.50,1.32) | 0.38 | 0.36 |
| **Smoke** |  |  |  |  |  |  |
| No | Ref. | 0.79(0.66, 0.94) | 0.01 | 0.45(0.35, 0.57) | <0.0001 | <0.0001 |
| Yes | Ref. | 0.89(0.67,1.17) | 0.40 | 0.97(0.57,1.64) | 0.91 | 0.72 |
| **CVD** |  |  |  |  |  |  |
| No | Ref. | 0.79(0.68, 0.93) | 0.01 | 0.52(0.40, 0.66) | <0.0001 | <0.0001 |
| Yes | Ref. | 1.11(0.61, 2.02) | 0.73 | 0.64(0.24, 1.67) | 0.35 | 0.44 |
| **preDM/DM** |  |  |  |  |  |  |
| No | Ref. | 0.76(0.55,1.04) | 0.09 | 0.57(0.39,0.83) | 0.004 | 0.004 |
| preDM | Ref. | 0.78(0.60,1.01) | 0.06 | 0.50(0.35,0.72) | <0.001 | <0.001 |
| DM | Ref. | 1.27(0.80,1.99) | 0.31 | 0.57(0.28,1.14) | 0.11 | 0.25 |

Each subgroup adjusted for all factors in model 2 except the stratification factor itself.

Abbreviations: DI-GM, dietary index for gut microbiota; T, tertile; MetALD: metabolic dysfunction and alcohol-associated liver disease; preDM, prediabetes mellitus; DM: diabetes mellitus; CVD, cardiovascular diseases.

β: Standardized Coefficients; CI: Confidence interval.

P for Trend: Tests for trends based on the variables containing the median values for each tertile.

Table S6 Association of DII Tertiles with MetALD stratified by variables of interest.

|  | **DII** | | | | | |
| --- | --- | --- | --- | --- | --- | --- |
|  | **T1** | **T2** | | **T3** | |  |
|  |  | **β (95%CI)** | **p** | **β (95%CI)** | **p** | **p for trend** |
| **Sex** |  |  |  |  |  |  |
| Male | Ref. | 1.52(1.20,1.91) | <0.001 | 1.79(1.35,2.36) | <0.0001 | <0.0001 |
| Female | Ref. | 1.27(0.94,1.73) | 0.12 | 1.40(1.01,1.95) | 0.04 | 0.05 |
| **Age** |  |  |  |  |  |  |
| < 65 yrs | Ref. | 1.37(1.08,1.74) | 0.01 | 1.39(1.08,1.80) | 0.01 | 0.01 |
| >= 65 yrs | Ref. | 0.48(0.25, 0.93) | 0.03 | 0.85(0.37, 1.94) | 0.70 | 0.68 |
| **Race** |  |  |  |  |  |  |
| Mexican American | Ref. | 0.99(0.66,1.48) | 0.96 | 1.54(1.01,2.34) | 0.05 | 0.07 |
| Other Race | Ref. | 1.37(0.87,2.14) | 0.17 | 2.34(1.37,4.00) | 0.002 | 0.003 |
| Non-Hispanic White | Ref. | 1.46(1.12,1.90) | 0.01 | 1.52(1.08,2.12) | 0.02 | 0.01 |
| Non-Hispanic Black | Ref. | 1.44(1.00,2.08) | 0.05 | 1.49(1.06,2.09) | 0.02 | 0.03 |
| **Smoke** |  |  |  |  |  |  |
| No | Ref. | 1.47(1.17,1.85) | 0.001 | 1.68(1.30,2.17) | <0.001 | <0.0001 |
| Yes | Ref. | 1.10(0.77,1.59) | 0.59 | 1.27(0.81,2.00) | 0.29 | 0.29 |
| **CVD** |  |  |  |  |  |  |
| No | Ref. | 1.38(1.13,1.70) | 0.002 | 1.55(1.25,1.94) | <0.001 | <0.0001 |
| Yes | Ref. | 1.75(0.93, 3.27) | 0.08 | 2.67(1.18, 6.04) | 0.02 | 0.02 |
| **preDM/DM** |  |  |  |  |  |  |
| No | Ref. | 1.69(1.24,2.32) | 0.001 | 2.19(1.53,3.14) | <0.0001 | <0.0001 |
| preDM | Ref. | 1.27(0.94,1.72) | 0.12 | 1.21(0.88,1.67) | 0.24 | 0.2 |
| DM | Ref. | 0.99(0.62,1.58) | 0.97 | 1.41(0.77,2.57) | 0.26 | 0.27 |

Each subgroup adjusted for all factors in model 2 except the stratification factor itself.

Abbreviations: DII: dietary inflammation index; T, tertile; MetALD: metabolic dysfunction and alcohol-associated liver disease; preDM, prediabetes mellitus; DM: diabetes mellitus; CVD, cardiovascular diseases.

β: Standardized Coefficients; CI: Confidence interval.

P for Trend: Tests for trends based on the variables containing the median values for each tertile.

Table S7 Association of different combinations of DI-GM and DII with MASLD stratified by variables of interest.

|  | **DII&DI-DM** | | | | | |
| --- | --- | --- | --- | --- | --- | --- |
|  | **Gut microbiota-unhealthy and pro-inflammatory** | **Composite diet category** | | **Gut microbiota-healthy and anti-inflammatory** | |  |
|  |  | **β (95%CI)** | **p** | **β (95%CI)** | **p** | **p for trend** |
| **Sex** |  |  |  |  |  |  |
| Male | Ref. | 1.88(1.35,2.62) | <0.001 | 2.74(1.87,3.99) | <0.0001 | <0.0001 |
| Female | Ref. | 2.08(1.37,3.14) | <0.001 | 2.51(1.57,4.01) | <0.001 | <0.001 |
| **Age** |  |  |  |  |  |  |
| < 65 yrs | Ref. | 2.16(1.67,2.79) | <0.0001 | 2.89(2.12,3.93) | <0.0001 | <0.0001 |
| >= 65 yrs | Ref. | 0.96(0.53,1.72) | 0.89 | 1.34(0.51,3.52) | 0.54 | 0.57 |
| **Race** |  |  |  |  |  |  |
| Mexican American | Ref. | 1.64(0.87,3.06) | 0.12 | 2.33(1.13,4.81) | 0.02 | 0.02 |
| Other Race | Ref. | 2.18(1.17,4.09) | 0.02 | 4.61(2.31,9.20) | <0.0001 | <0.0001 |
| Non-Hispanic White | Ref. | 2.04(1.50,2.78) | <0.0001 | 2.59(1.71,3.94) | <0.0001 | <0.0001 |
| Non-Hispanic Black | Ref. | 1.22(0.72,2.05) | 0.45 | 1.43(0.77,2.65) | 0.25 | 0.25 |
| **Smoke** |  |  |  |  |  |  |
| No | Ref. | 2.24(1.68,2.99) | <0.0001 | 3.00(2.08,4.34) | <0.0001 | <0.0001 |
| Yes | Ref. | 0.99(0.55,1.77) | 0.96 | 1.35(0.68,2.68) | 0.39 | 0.2 |
| **CVD** |  |  |  |  |  |  |
| No | Ref. | 1.99(1.53,2.57) | <0.0001 | 2.65(1.95,3.61) | <0.0001 | <0.0001 |
| Yes | Ref. | 1.33(0.53,3.35) | 0.54 | 2.13(0.73,6.22) | 0.16 | 0.13 |
| **preDM/DM** |  |  |  |  |  |  |
| No | Ref. | 1.74(1.13,2.67) | 0.01 | 2.65(1.66,4.22) | <0.0001 | <0.0001 |
| preDM | Ref. | 2.14(1.48,3.11) | <0.001 | 2.60(1.67,4.06) | <0.0001 | <0.0001 |
| DM | Ref. | 1.57(0.72,3.46) | 0.26 | 1.81(0.72,4.53) | 0.20 | 0.2 |

Each subgroup adjusted for all factors in model 2 except the stratification factor itself.

Abbreviations: DI-GM, dietary index for gut microbiota; DII: dietary inflammation index; MetALD: metabolic dysfunction and alcohol-associated liver disease; preDM, prediabetes mellitus; DM: diabetes mellitus; CVD, cardiovascular diseases.

β: Standardized Coefficients; CI: Confidence interval.

P for Trend: Tests for trends based on the variables containing the median values for each tertile.
